# Biological activity and quantitative determination of free amino acids from muskmelon (*Cucumis melo* L.) extracts

**DOI:** 10.3389/fphar.2026.1822910

**Published:** 2026-05-29

**Authors:** Abdel Moneim Elhadi Sulieman, Salwa M. Elamin, Mamdouh Alshammari, Fahad Almarshidi, Safa Mustafa Ibrahim, Zakaria Ahmed Saleh, Hattim Makki Mohamed, Riadh Badraoui

**Affiliations:** 1 Department of Biology, College of Science, University of Ha’il, Ha’il, Saudi Arabia; 2 Department of Biology, College of Science, King Khalid University, Abha, Saudi Arabia; 3 College of Health and Health Informatics, University of Ha’il, Ha’il, Saudi Arabia; 4 Research and Training Station, King Faisal University, Al-Ahsa, Saudi Arabia; 5 Date Palm Research Center of Excellence, King Faisal University, Al-Ahsa, Saudi Arabia; 6 Section of Histology-Cytology, Faculty of Medicine, University of Tunis El Manar, Tunis, Tunisia

**Keywords:** amino acids, antimicrobial activity, Cucumis melo, food valorization, GC–MS, molecular docking, phytochemicals

## Abstract

**Introduction:**

Muskmelon (*Cucumis melo L.*, family *Cucurbitaceae*) processing generates substantial quantities of underutilized by-products, including peel/rind, stems, and leaves, which may serve as valuable sources of bioactive compounds. This study aimed to evaluate the phytochemical composition, quantify free amino acids, assess antimicrobial activity, and investigate molecular interactions of these by‐products to support their potential for food and pharmaceutical valorization.

**Methods:**

Phytochemical screening and thin‐layer chromatography (TLC) were employed to identify major secondary metabolites. Free amino acids were quantified using gas chromatography–mass spectrometry (GC–MS). Antimicrobial activity of methanolic extracts from different plant parts was evaluated against *Staphylococcus aureus, Bacillus subtilis, Escherichia coli*, and *Candida albicans* using the agar well diffusion method. Molecular docking analysis was performed to assess interactions between identified amino acids and target proteins, including *S. aureus* tyrosyl‐tRNA synthetase (PDB: 1JIJ) and *C. albicans* Sap1 protease (PDB: 2QZW).

**Results:**

Phytochemical analysis confirmed the presence of sterols, triterpenes, flavonoids, phenolics, saponins, and carotenoids, with peel/rind and leaves showing the richest profiles. GC–MS analysis revealed glutamine (28.0%), alanine (11.2%), and aspartic acid (9.3%) as the predominant free amino acids. Methanolic extracts exhibited significant, concentration‐dependent antimicrobial activity, with stronger inhibition observed against Gram‐positive bacteria and *C. albicans*. Molecular docking demonstrated favorable binding affinities of selected amino acids toward target enzymes, supporting the observed biological activity.

**Discussion:**

The findings highlight muskmelon by‐products as promising sources of bioactive compounds with notable antimicrobial potential. While amino acids demonstrated favorable in silico interactions, the antimicrobial activity is primarily attributed to phytochemical constituents. These results support the potential application of muskmelon waste in functional food development and natural antimicrobial formulations. Further studies, including MIC/MBC determination and phytochemical‐targeted docking, are recommended to validate and expand these findings.

## Graphical Abstract

**Figure:**
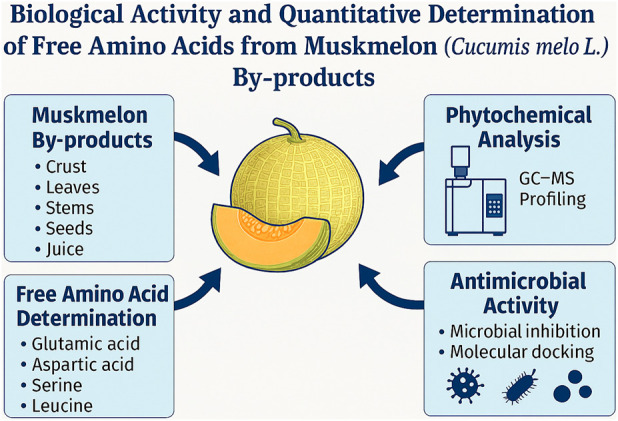

## Introduction

Muskmelon (Cucumis melo L.), a member of the Cucurbitaceae family, is grown a lot in tropical and subtropical areas because it is good for you, tastes well, and has medicinal benefits. The fruit is full of important nutrients including potassium, vitamin C, β-carotene, and dietary fiber, which help it fight free radicals, protect the heart, and keep you hydrated. In addition to its nutritional benefits, C. melo has been used for a long time to treat high blood pressure, digestive problems, and kidney problems (Yadav et al., 2025; Soh et al., 2024). Recent progress in phytochemical and pharmacological research has made it even clearer that Cucurbitaceae species have therapeutic potential. These plants are known to be rich sources of biologically active chemicals that have a wide range of health advantages (Ale et al., 2026).

People have been paying more attention to the phytochemical richness and functional qualities of Cucumis melo and similar plants in the last several years. Peels, seeds, leaves, and stems of different plants have been revealed to have a lot of secondary metabolites, such as phenolics, flavonoids, sterols, triterpenes, and cucurbitacins. These substances are recognized for their substantial biological activities, encompassing antioxidant, antibacterial, anti-inflammatory, and anticancer properties (Mechchate et al., 2021). Importantly, new research shows that the phytochemical makeup and bioactivity of different plant tissues can be very varied. This is because they have different metabolic pathways and adaptive defensive systems (da Silva and Clerici, 2025). This diversity highlights the necessity of properly assessing underused plant components to fully harness their bioactive potential.

The increasing global apprehension regarding antimicrobial resistance (AMR) has amplified the pursuit of alternative and sustainable antimicrobial agents sourced from natural origins. Phytochemicals derived from plants have garnered significant attention owing to their extensive antibacterial properties, diminished toxicity, and a reduced propensity to provoke resistance in contrast to traditional antibiotics. These bioactive molecules can kill germs in a number of ways, such as by breaking down microbial cell membranes, blocking important enzymatic pathways, and causing oxidative stress (Mechchate et al., 2021). In this perspective, agricultural by-products and food processing wastes constitute an unexploited source of significant bioactive chemicals with prospective applications in pharmacology, food preservation, and nutraceutical development. Processing fruits and vegetables around the world creates a lot of waste, often more than 30% of total production. This costs money and hurts the environment (FAO, 2019). Processing muskmelons creates a lot of waste that cannot be eaten, like peels, stems, and leaves. These are often thrown away even though they have a lot of phytochemicals in them. Recent research has underscored the significance of valorizing agro-industrial by-products within a circular bioeconomy framework, converting waste materials into high-value functional ingredients for medicinal and food uses (Khalil et al., 2025). This method not only makes things more sustainable, but it also helps make bioactive substances that are good for the environment and cheap. Modern pharmacological research increasingly incorporates computational methodologies, like molecular docking and molecular dynamics simulations, to forecast interactions between bioactive chemicals and biological targets, alongside experimental approaches. These *in silico* techniques facilitate expedited screening, mechanistic elucidation, and the prioritizing of prospective therapeutic candidates, hence expediting the drug development process (Babalola et al., 2025; Natesan et al., 2025). Nonetheless, it is crucial to acknowledge that computational predictions yield just first insights and should be interpreted judiciously with experimental results, especially when addressing intricate phytochemical combinations.

This study intends to thoroughly assess the biological potential of muskmelon by-products, specifically the peel/rind (SP4a), stem (SP4b), and leaves (SP4c), within an integrated framework. This study aims to: (i) characterize the phytochemical composition via qualitative screening and thin-layer chromatography (TLC); (ii) ascertain the free amino acid profile through GC–MS analysis; (iii) evaluate the *in vitro* antimicrobial activity against specific bacterial and fungal pathogens; and (iv) investigate potential molecular interactions of identified amino acids with key microbial enzymes through molecular docking. This study integrates experimental and computational methodologies, establishing a solid foundation for the valorization of muskmelon waste as a potential source of natural antibacterial and functional chemicals.

## Materials and methods

### Plant material and extraction

Dried powders (25 g) of peel/rind (SP4a), stem (SP4b), and leaves (SP4c) were sequentially extracted using petroleum ether (60 °C–80 °C), methanol (99.8%), and distilled water. Extraction was performed by maceration (24 h, room temperature) with intermittent shaking. Extracts were filtered and concentrated using a rotary evaporator under reduced pressure and stored at 4 °C (Cao et al., 2025).

### Thin layer chromatography (TLC)

TLC was performed on silica gel 60 F254 plates. Extracts were developed using solvent systems including:

- Hexane:ethyl acetate (7:3)
- chloroform: methanol (9:1)

Spots were visualized under UV light (254 and 366 nm) and by spraying with vanillin–sulfuric acid reagent followed by heating. Retention factor (Rf) values were calculated.

### GC–MS analysis of amino acids

GC–MS analysis was performed using a gas chromatograph coupled to a mass spectrometer equipped with an HP-5MS column (30 m × 0.25 mm, 0.25 μm film thickness).

### Operating conditions

- Carrier gas: Helium (1 mL/min)
- Injection volume: 1 µL (split ratio 10:1)
- Oven temperature program: 60 °C (2 min), increased to 280 °C at 10 °C/min
- Ionization: Electron impact (70 eV)

Identification was performed by comparison with standard spectra from NIST library.

### Antimicrobial activity assay

The antimicrobial activity of the plant extracts was evaluated using the agar well diffusion method (Sulieman et al., 2024). Microbial suspensions were adjusted to 1 × 10^6^ CFU/mL and spread onto Mueller–Hinton agar (bacteria) or Sabouraud dextrose agar (fungi) plates. Wells (10 mm diameter) were bored aseptically into the agar, and 100 µL of each extract (10–1000 μg/mL, prepared in DMSO) were added. Plates were allowed to stand at room temperature for 1 h to facilitate diffusion, then incubated at 37 °C for 24 h (bacteria) or 28 °C for 24–48 h (C. albicans). Zones of inhibition were measured in millimeters. DMSO served as a negative control, whereas, Gentamicin (10 μg/mL) and penicillin (10 μg/mL) were used as positive controls for bacteria, while mycostatin (100 IU/mL) and copper sulfate (1 mg/mL) were used for fungi.

### Statistical analysis

All experiments were performed in triplicate (n = 3), and results were expressed as mean ± SD. Statistical differences were analyzed using one-way ANOVA followed by Tukey’s *post hoc* test at p < 0.05.”

### Computational modeling: Affinity and molecular interactions

The 3D chemical structures of the different identified amino acids were collected from PubChem databases, while the 3D crystal structures of Staph. aureus* tyrosyl-tRNA synthetase (PDB ID: 1JIJ) and the secreted aspartic proteinase (Sap) 1 from *C. albicans* (PDB ID: 2QZW) were retrieved from RCSB databases. Both amino acids and macromolecules were prepared for minimization as previously described (Ventola, 2015; Cowan, 1999; Badraoui et al., 2025). Polar hydrogens and Kollman charges were added, while heteroatoms and crystallized water molecules were removed. The amino acids and macromolecules were saved in “pdbqt” format and used to assess the binding affinities and explore the molecular interactions based on the CHARMm force field (Mirabella et al., 2014; Cowan, 1999; Borges et al., 2013) using vina software packages, discovery studio visualizer and Pymol as previously reported (Almuzaini et al., 2024; Lakhrem et al., 2024; Boudjema et al., 2025). The dimensions X, Y and Z of the grid box have been maintained as those of the native ligands.

## Results

### Phytochemical screening

A wide variety of secondary metabolites were found in muskmelon by-products, according to qualitative phytochemical study (Table 1). The peel/rind (SP4a) and leaves (SP4c) included an abundance of sterols, triterpenes, carotenoids, reducing sugars, coumarins, saponins, and proteins, but the stem (SP4b) had a lower concentration of these phytochemicals. The peel/rind and leaves consistently contained tannins, flavonoids, and phenolic compounds, whereas the stem either did not contain any of these substances or only had very little levels. No sample tested positive for alkaloids or sesquiterpene lactones. The greatest variety of phytochemicals, including terpenoids, saponins, phenolics, and flavonoids, was found in the peel/rind (SP4a) and the leaves (SP4c). The phytochemical content of stem (SP4b) was relatively lower. These findings point to the peel/rind and leaves as the parts of the plant with the highest concentrations of bioactive substances.

**TABLE 1:** Qualitative phytochemical analysis of muskmelon peel/rind (SP4a), stem (SP4b), and leaves (SP4c). Abundance levels are expressed as follows: + (low), ++ (medium), +++ (high), and – (absent).

Compound class	SP4a (peel/rind)	SP4b (Stem)	SP4c (Leaves)
Sterols & triterpenes	+	+	+
Carotenoids	–	+	+++
Reducing compounds	+++	–	+++
Saponins	+	±	+++
Carbohydrates	++	++	–

### Thin-layer chromatography (TLC) profiling

**FIGURE 1:**
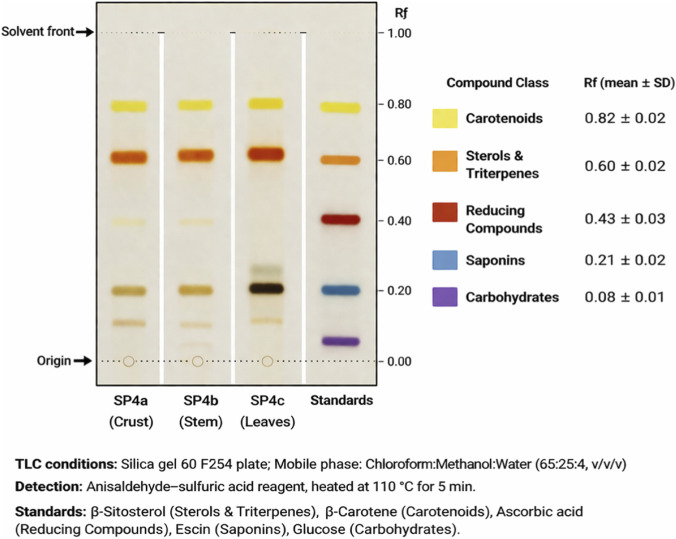
TLC chromatogram of extracts from *S. platyphylla* (SP4a, SP4b, and SP4c) and standards. Bands were visualized with anisaldehyde-sulfuric acid reagent and Rf values were calculated relative to the solvent front. The observed bands correspond to carotenoids, sterols and triterpenes, reducing compounds, saponins, and carbohydrates.

TLC analysis corroborated the results of the preliminary phytochemical screening and revealed clear qualitative differences among the three plant parts. The developed chromatogram (Figure 1) showed well-resolved bands with distinct *Rf* values, indicating the presence of multiple classes of phytoconstituents.

SP4a (crust/peel) exhibited the most complex chromatographic profile, with several intense and well-defined bands distributed across a wide *Rf* range. Prominent bands corresponding to carotenoids (*Rf* ≈ 0.82), sterols and triterpenes (*Rf* ≈ 0.60), reducing compounds (*Rf* ≈ 0.43), saponins (*Rf* ≈ 0.21), and carbohydrates (*Rf* ≈ 0.08) were observed, reflecting a high diversity of bioactive metabolites.

SP4c (leaves) showed a comparable pattern, with bands appearing at similar *Rf* positions; however, the intensity and number of bands were slightly lower than those of SP4a, suggesting moderate phytochemical richness.

In contrast, SP4b (stem) displayed fewer and less intense bands, indicating a relatively simpler chemical composition and lower abundance of detectable compounds.

Overall, the TLC chromatogram confirms that the peel/rind contains the highest diversity and concentration of phytochemicals, followed by leaves, while the stem possesses a more limited phytochemical profile. The distinct *Rf* patterns further support the presence of chemically diverse constituents in different plant parts.

### Amino acid composition by GC–MS

**FIGURE 2:**
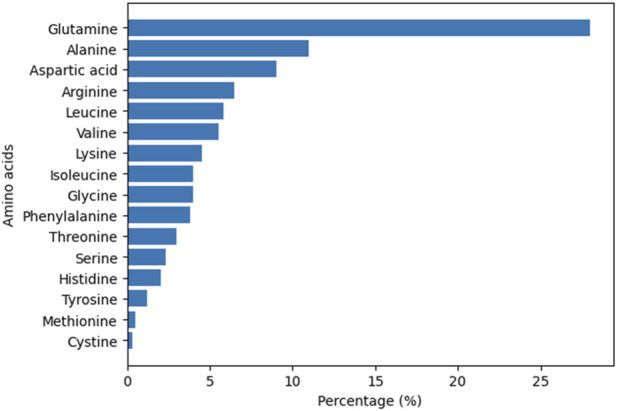
Amino acid composition of *Cucumis melo* peel/rind (SP4a) determined by GC–MS analysis. Data are expressed as a percentage (%) of the total detected amino acids.

GC–MS analysis of free amino acids revealed that the muskmelon peel/rind contains several essential and non-essential amino acids at varying concentrations (Table 2; Figure 2). Glutamine was the dominant amino acid (28.0%), followed by alanine (11.2%), aspartic acid (9.3%), serine (6.7%), and valine (6.5%). Essential amino acids including leucine, phenylalanine, threonine, and isoleucine were also present. This composition suggests high nutritional value and possible applications in functional foods or dietary supplements.

**TABLE 2:** Amino acid content of muskmelon peel/rind (SP4a).

Amino acid	Amount (mg)	Percentage (%)
Glutamine	134.37	28.0
Alanine	53.96	11.2
Aspartic acid	44.82	9.3
Arginine	30.81	6.4
Leucine	27.21	5.7
Valine	26.24	5.5
Lysine	21.48	4.5
Isoleucine	19.27	4.0
Glycine	19.29	4.0
Phenylalanine	18.06	3.8
Threonine	14.39	3.0
Serine	10.97	2.3
Histidine	9.48	2.0
Tyrosine	5.85	1.2
Methionine	2.36	0.5
Cystine	1.55	0.3

### Antimicrobial activity of extracts

**FIGURE 3:**
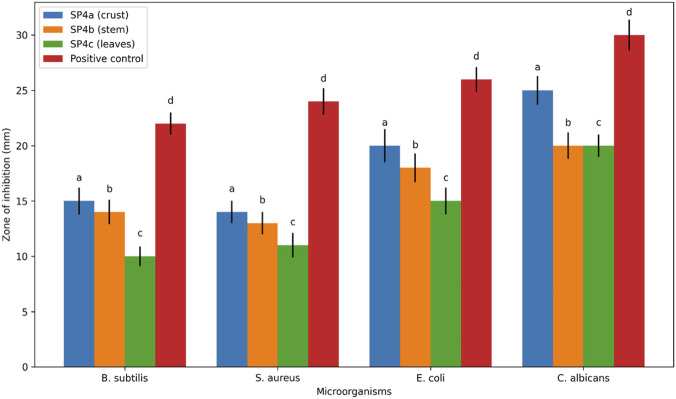
Antimicrobial activity of methanolic extracts from muskmelon parts (SP4a, SP4b, SP4c) against selected microorganisms. Data represent inhibition zone diameters (mm) at 1000 μg/mL (mean ± SD, n = 3). Different letters indicate significant differences (*p* < 0.05).

The antimicrobial activities of the methanolic extracts obtained from muskmelon peel/rind (SP4a), stem (SP4b), and leaves (SP4c) were assessed using the well diffusion method (Figure 3). Among the tested samples, SP4a and SP4c produced the largest inhibition zones, demonstrating strong and concentration-dependent antimicrobial effects against *Bacillus subtilis*, *Staphylococcus aureus*, and *Candida* albicans. Inhibition increased steadily from 10 to 1000 μg/mL, with the most pronounced activity observed at the highest concentration. In contrast, SP4b exhibited only weak to moderate inhibition across all tested microorganisms, although its activity also increased significantly with concentration (p < 0.05).

All extracts showed limited activity against *Escherichia coli*, consistent with the well-known reduced susceptibility of Gram-negative bacteria due to the protective outer membrane barrier. Overall, SP4a and SP4c displayed notable antimicrobial potential, with inhibition zones at 1000 μg/mL approaching those produced by standard antibiotic controls. Although inhibition zone analysis demonstrated strong antimicrobial activity, future work will include determination of MIC and MBC values to quantify bacteriostatic and bactericidal effects.”

### Determination of inhibition zones

At 1000 μg/mL, the peel/rind extract produced the highest inhibition zone against *B. subtilis* (24.2 ± 0.8 mm) and *C. albicans* (19.3 ± 0.6 mm, followed closely by the leaf extract. Both extracts were moderately effective against *S. aureus*. Copper (II) sulfate showed superior antifungal activity, whereas mycostatin exhibited lower inhibition. DMSO did not inhibit any microorganism, confirming its suitability as a negative control (Table 3).

**TABLE 3:** Antimicrobial activity of muskmelon extracts and controls expressed as inhibition zone diameter (mm) at 1000 μg/mL (mean ± SD, n = 3).

Test sample	*B. subtilis* (mm)	*S. aureus* (mm))	*E.coli*	*C. albicans* (mm)
SP4a	24.2 ± 0.8	18.6 ± 0.5	10.2 ± 0.4	19.3 ± 0.6
SP4b	12.4 ± 0.5	10.9 ± 0.4	8.3 ± 0.3	11.6 ± 0.5
SP4c	22.9 ± 0.7	17.8 ± 0.6	9.7 ± 0.4	18.8 ± 0.7
Gentamicin	28.5 ± 0.6	26.7 ± 0.5	24.9 ± 0.6	-
Penicillin	26.3 ± 0.7	24.5 ± 0.6	20.2 ± 0.5	-
CuSO_4_	-	-	-	25.6 ± 0.8
Mycostatin	-	-	-	21.4 ± 0.6
DMSO	0	0	0	0

Values represent inhibition zone diameters at 1000 μg/mL. Peel/rind and leaf extracts showed clear concentration-dependent antimicrobial activity, confirming the presence of biologically active constituents. DMSO, exhibited no inhibition, validating its role as a negative control.

The clear concentration-dependent inhibition confirmed that antimicrobial constituents are present in measurable and biologically active quantities in the peel/rind and leaves.

### Computational analyses and molecular interactions assay

**FIGURE 4:**
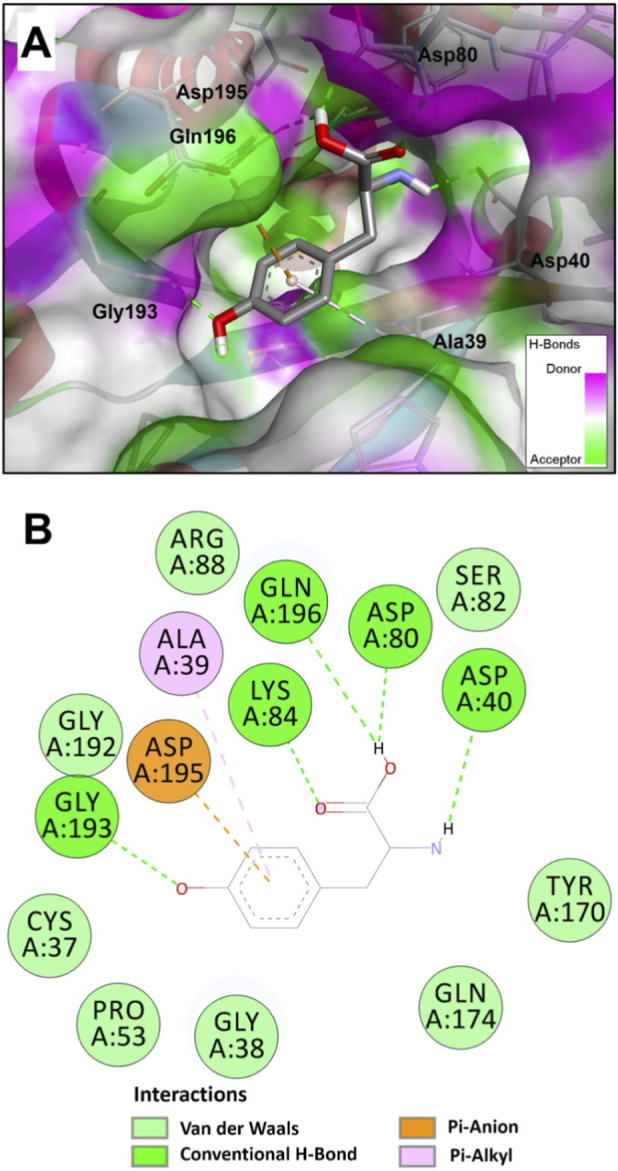
**(A)** 3D illustration of tyrosine while complexed with *Staph. aureus* tyrosyl-tRNA synthetase (pdb id: 1JIJ) and **(B)** the resulted diagram of interactions.

**FIGURE 5:**
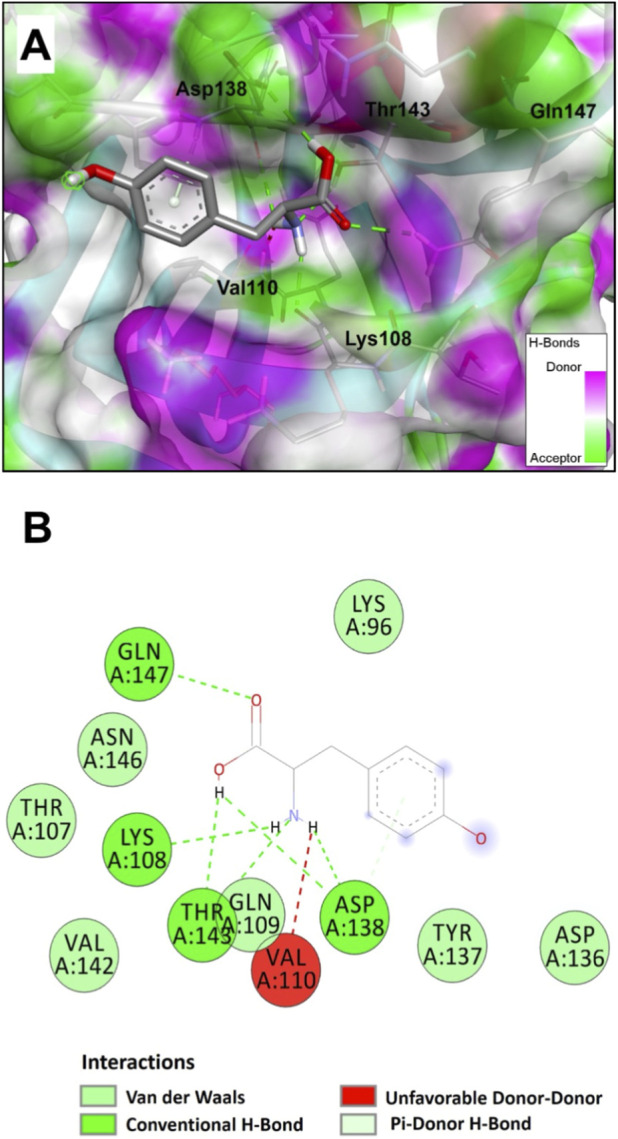
**(A)** 3D illustration of tyrosine while complexed with the Secreted aspartic proteinase (Sap) 1 from *C. albicans* (pdb id: 2QZW) and **(B)** the resulted diagram of interactions.

Table 4 exhibits that the identified amino acids possessed negative affinities and acceptable root mean square deviation (RMSD). The binding affinities reached −6.7 and −6.1 kcal/mol, specifically for tyrosine while complexed with 1JIJ and 2QZW, respectively. While tyrosine possessed the best binding scores, arginine and histidine were predicted to establish the highest number (n = 7) of conventional H-bonds once complexed with each of 1JIJ and 2QZW. The molecular interactions of arginine included Tyr170, Asp40, Thr75, Asp177, Asp40, Asp80, and Gln196 associated with some others such as salt bridge: attractive charge and attractive charge and carbon H-bond (Table 5; Figure 4). However, histidine interacted with Asp32, Asp218, Asp218, Asp32, Gly220, Thr221, Gly220 (Table 5; Figure 5). While complexed with 1JIJ, Arginine exhibited the deepest embedding that reached 1.881 Å only. Furthermore, phenylalanine showed the deepest embedding (1.947 Å only), while complexed with 2QZW. The computational modeling results of the current study supported the experimental findings and lend support to the beneficial effects of muskmelon.

**TABLE 4:** Binding affinity and root mean square deviation (RMSD) of the identified amino acids and the targeted receptors; *Staph. aureus* tyrosyl-tRNA synthetase and the Secreted aspartic proteinase (Sap) 1 from *C. albicans* (pdb id: 1JIJ and 2QZW, respectively).

Entry	Binding affinity (kcal/mol)		RMSD (lower-upper)	
	1JIJ	2QZW	1JIJ	2QZW
Alanine	−4.6	−3.7	0.0–4.26	0.0–38.66
Arginine	−6.4	−5.0	0.0–4.82	0.0–16.64
Aspartic acid	−5.8	−4.9	0.0–5.47	0.0–34.48
Cystine	−6.1	−4.4	0.0–13.91	0.0–37.79
Glutamine	−6.0	−4.7	0.0–4.21	0.0–24.38
Glycine	−4.0	−3.9	0.0–27.07	0.0–25.66
Histidine	−6.4	−5.2	0.0–5.27	0.0–27.56
Lysine	−5.3	−4.4	0.0–26.31	0.0–19.97
Methionine	−5.0	−4.2	0.0–4.61	0.0–23.90
Phenylalanine	−6.5	−6.1	0.0–27.45	0.0–24.68
Serine	−4.6	−4.1	0.0–3.91	0.0–34.60
Threonine	−5.0	−4.3	0.0–4.50	0.0–34.17
Tyrosine	−6.7	−6.1	0.0–23.62	0.0–25.06
Valine	−5.2	−4.5	0.0–26.17	0.0–23.67
Isoleucine	−4.7	−4.8	0.0–25.34	0.0–23.71
Leucine	−5.4	−4.8	0.0–26.59	0.0–24.12

**TABLE 5:** Number of conventional H-bonds, closest interacting residues and distance to closest interacting residue (Å) of the identified amino acids and the targeted receptors; *Staph. aureus* tyrosyl-tRNA synthetase and the Secreted aspartic proteinase (Sap) 1 from *C. albicans* (pdb id: 1JIJ and 2QZW, respectively).

Saccharide	No. Conventional H-Bond	Closest Interacting residues		
		Interaction categories/Residues	Closest residue (distance, Å)	No. closest interacting residues
*Staph. aureus* tyrosyl-tRNA synthetase (1JIJ)				
Tyrosine	5	Conventional H-Bond: Lys84, Gly193, Asp40, Asp80, Gln196 π-Anion: Asp195 π-Alkyl: Ala39	Asp80:OD2 (2.123)	7
Phenylalanine	5	Conventional H-Bond: Asp40, Asp80, Gln196, Asp80, Gln196 π-Alkyl: Leu70	Gln196:OE1 (2.093)	4
Arginine	7	Salt Bridge; Attractive charge: Asp40 Attractive charge: Asp80 Conventional H-Bond: Tyr170, Asp40, Thr75, Asp177, Asp40, Asp80, Gln196 Carbon H-Bond: Gly38	Asp177:OD1 (1.881)	7
Secreted aspartic proteinase (Sap) 1 from *C. albicans* (2QZW)				
Tyrosine	6	Conventional H-Bond: Thr143, Gln147, Asp138, Lys108, Asp138, Thr143 π-Donor H-bond: Asp138	Lys108:O (2.176)	4
Phenylalanine	4	Conventional H-Bond: Thr143, Asp138, Lys108, Asp138 π-Donor H-bond: Gln147 π-Alkyl: Lys108	Asp138:O (1.947)	4
Histidine	7	Conventional H-Bond: Asp32, Asp218, Asp218, Asp32, Gly220, Thr221, Gly220	Gly220:O (2.142)	4

## Discussion

### Phytochemical composition and biological relevance

The present study demonstrates that muskmelon by-products, particularly peel/rind (SP4a) and leaves (SP4c), are rich in diverse classes of phytochemicals, including phenolics, flavonoids, sterols, triterpenes, and saponins. These findings are consistent with previous reports highlighting the Cucurbitaceae family as a valuable source of bioactive compounds with multifunctional biological activities (Mechchate et al., 2021; Ale et al., 2026). The higher phytochemical abundance observed in peripheral tissues such as peel and leaves can be attributed to their protective roles against environmental stressors, pathogens, and herbivores, leading to enhanced biosynthesis of defensive secondary metabolites.

The qualitative and chromatographic analyses revealed a complex chemical profile, suggesting the presence of synergistic interactions among multiple compounds. Such synergism is well-documented in plant extracts and is often responsible for enhanced biological activity compared to isolated compounds. Phenolic and flavonoid compounds, in particular, are known to contribute significantly to antimicrobial and antioxidant properties through mechanisms involving membrane disruption, enzyme inhibition, and redox imbalance (Mechchate et al., 2021). These findings support the hypothesis that muskmelon waste materials are not merely agricultural residues but represent a promising source of functional bioactive compounds.

### Antimicrobial activity and mechanistic insights

The antimicrobial results clearly indicate that SP4a and SP4c exhibited significant, concentration-dependent inhibitory activity against Gram-positive bacteria (*Staphylococcus aureus*, *Bacillus subtilis*) and the fungal pathogen *Candida albicans*, while showing comparatively weaker activity against Gram-negative *Escherichia coli*. This differential susceptibility is consistent with the structural differences between Gram-positive and Gram-negative bacteria, particularly the presence of an outer membrane in Gram-negative species that restricts the penetration of bioactive compounds.

The observed antimicrobial activity can be attributed primarily to the presence of phenolics, flavonoids, and saponins. These compounds exert their effects through multiple complementary mechanisms, including disruption of microbial cell membranes, interference with nucleic acid synthesis, inhibition of key metabolic enzymes, and induction of oxidative stress. The stronger activity observed in peel/rind and leaves correlates well with their higher phytochemical content, reinforcing the role of these compounds as key contributors to antimicrobial efficacy.

It is important to emphasize that the antimicrobial activity observed in this study reflects the combined effect of complex phytochemical mixtures rather than individual constituents. Therefore, the results highlight the importance of considering whole-extract activity when evaluating plant-derived antimicrobials. Additionally, the quantitative inhibition zone data (Table 3) confirm statistically significant differences among extracts (p < 0.05), further supporting the reproducibility and biological relevance of the findings.

However, a major limitation of the present study is the absence of minimum inhibitory concentration (MIC) and minimum bactericidal concentration (MBC) determinations. While the agar well diffusion method provides valuable preliminary screening, it does not allow precise quantification of antimicrobial potency or differentiation between bacteriostatic and bactericidal effects. Therefore, future studies should incorporate broth dilution assays to determine MIC and MBC values, enabling more accurate pharmacological evaluation and comparison with standard antimicrobial agents.

### Amino acid profile and functional Implications

The GC–MS analysis revealed that muskmelon peel/rind contains a diverse profile of free amino acids, with glutamine, alanine, and aspartic acid being the predominant components. These amino acids play essential roles in metabolic regulation, immune function, and nutritional supplementation. For instance, glutamine is a key substrate for rapidly proliferating cells and is critical for maintaining intestinal integrity and immune responses, while alanine is involved in glucose metabolism and energy homeostasis.

Despite their biological importance, it is important to clarify that amino acids are not typically considered primary antimicrobial agents. Their inclusion in the present study was based on their abundance and potential interaction with microbial targets in computational analyses. The nutritional and functional significance of these amino acids, however, supports the potential application of muskmelon by-products in functional foods, dietary supplements, and nutraceutical formulations.

### Molecular docking: Insights and limitations

Molecular docking analysis revealed that several amino acids exhibited moderate binding affinities (approximately −4.0 to −6.7 kcal/mol) toward selected microbial targets, including *Staphylococcus aureus* tyrosyl-tRNA synthetase (1JIJ) and *Candida albicans* Sap1 protease (2QZW). Among the tested compounds, tyrosine, arginine, and histidine demonstrated relatively stronger interactions, forming multiple hydrogen bonds and stabilizing interactions within the active sites.

While these findings provide useful preliminary insights into potential molecular interactions, it is critical to interpret them cautiously. The observed binding affinities fall within the range of moderate interactions and do not indicate strong inhibitory activity. Moreover, the antimicrobial effects observed experimentally are more likely driven by complex phytochemical mixtures rather than individual amino acids. Therefore, the docking results should be considered as exploratory and hypothesis-generating rather than confirmatory.

Future studies should focus on docking and molecular dynamics simulations of key phytochemicals such as flavonoids, phenolic acids, and cucurbitacins, which are more directly associated with antimicrobial activity. Additionally, integrating *in silico* results with experimental validation (e.g., enzyme inhibition assays) would provide a more robust mechanistic understanding.

### Valorization potential and circular bioeconomy perspective

The findings of this study strongly support the valorization of muskmelon by-products within a circular bioeconomy framework. Instead of being discarded as waste, peel/rind and leaves can be repurposed as valuable sources of natural antimicrobial and functional compounds. Potential applications include their use as natural food preservatives, active packaging components, and nutraceutical ingredients.

The integration of such plant-derived bioactive compounds into food and pharmaceutical systems aligns with current trends toward sustainable, eco-friendly, and health-promoting solutions. Moreover, the utilization of agricultural waste contributes to waste reduction, resource efficiency, and economic value generation. These aspects are particularly relevant in the context of global sustainability goals and increasing demand for natural alternatives to synthetic additives.

## Conclusion

Muskmelon (Cucumis melo L.) by-products’ phytochemical composition, amino acid profile, antibacterial activity, and molecular interactions are examined in this study. Peel/rind and leaves contain bioactive chemicals, mainly phenolics and flavonoids, that have strong antibacterial activity against Gram-positive bacteria and *Candida* albicans.

Combining experimental and computational methods reveals the biological potential of underutilized plant components. Antimicrobial activity is mostly due to complex phytochemical elements, not amino acids, therefore docking data should be considered as preliminary. Future studies should address the lack of MIC and MBC data.

This study shows that muskmelon by-products can be used in nutraceutical, pharmaceutical, and food preservation systems as sustainable sources of natural antibacterial and functional chemicals. The practical use of phytochemical isolation, mechanism validation, and improved pharmacological evaluation requires more investigation.

## Data Availability

The data that supports the findings of this study are available on request from the corresponding author on reasonable request.
